# Comparison of elastic stable intramedullary nailing versus retrograde screw fixation for pubic ramus fractures—a biomechanical study

**DOI:** 10.1007/s00068-025-02791-0

**Published:** 2025-03-08

**Authors:** Julian Scherer, Yasmin Youssef, Toni Wendler, Benjamin Fischer, Stefan Schleifenbaum, Georg Osterhoff

**Affiliations:** 1 Orthopaedic Research Unit, Division of Orthopaedic Surgery, Groote Schuur Hospital, University of Cape Town, Cape Town, South Africa; 2 Department of Traumatology, University Hospital of Zurich, Zurich, Switzerland; 3 Department of Orthopaedics, Trauma and Plastic Surgery, University Hospital Leipzig, Leipzig, Germany; 4 Centre for Research on Musculoskeletal Systems, Leipzig University, Leipzig, Germany

**Keywords:** Pelvis, Fracture, Pubic bone, Screw fixation, Intramedullary nail, Biomechanical

## Abstract

**Background:**

Percutaneous minimally invasive retrograde screw fixation is a widely accepted treatment strategy for patients with superior ramus fractures and has shown good biomechanical stability compared to plating. Recently, elastic stable intramedullary nailing (ESIN) devices have been proposed as an alternative in the treatment of superior ramus fractures. However, biomechanical studies on this new treatment are lacking. Thus, the aim of this study was to compare the biomechanical stability of ESIN in pubic ramus fractures versus retrograde screw fixation.

**Methods:**

Standardized pubic ramus fractures (Nakatani type II) were created in fresh-frozen paired hemipelves. Fractures were either stabilized with a 6.5 mm cannulated screw (n = 4) or a 3.5 mm Stainless Steel Elastic Nail System (n = 4). In a validated setup, a cyclic loading protocol was applied with increasing axial force (1500 cycles, 250–750 N). Outcome parameters were fracture mobility over time, fracture displacement and construct survival. Descriptive and opto-metric methods were used to describe the mode of failure.

**Results:**

Amongst all tested hemipelves (n = 8), no construct failure was observed. There was no significant difference in mean vertical fracture displacement between the groups (ESIN 0.07 mm, SD 0.12 versus screw 0.04 mm, SD 0.05; p = 0.773). After 500 cycles at 250 N, mean vertical fracture displacement was 0.09 mm (SD 0.16) in the ESIN group and 0.03 mm (SD 0.04) in the screw group (p = 0.773). After subsequent 500 cycles at 500 N in the vertical plane, mean fracture displacement increased to 0.35 mm (SD 0.31) in the ESIN group and to 0.14 mm (SD 0.17) in the screw group (p = 0.281). With a maximum load of 750 N, after 500 cycles, mean fracture displacement was 0.58 mm (SD 0.51) in the ESIN group and 0.31 mm (SD 0.26) in the screw group (p = 0.376). There was no difference between the implants regarding the accumulated fracture movement over time (ESIN 494 mm*cycles, SD 385 versus screw 220 mm*cycles, SD 210; p = 0.259).

**Conclusions:**

In this in-vitro biomechanical study, fixation of superior ramus fracture using ESIN was not different in construct survival, relative motion to fracture, and fracture displacement when compared to retrograde screw fixation.

## Introduction

Pubic ramus fractures are commonly observed in both high-energy and low-energy trauma. [1] The incidence of pubic ramus fractures lies between 6.9/100,000 per year and 25.6/100,000 per year for the elderly population. [2] Therefore, these fractures pose a significant clinical and socioeconomic challenge. Literature indicates that conservative treatment of superior pubic ramus fractures is inferior to surgical fixation, especially in significantly displaced fractures. [3] Traditionally, plating of superior ramus fractures is the treatment gold-standard. [4] Due to the invasiveness, longer operation duration and potential complications such as bleeding of this open reduction and internal fixation (ORIF), there was a shift towards closed reduction and internal fixation (CRIF) using percutaneous retrograde pubic ramus screws for the operative treatment of unstable superior ramus fractures. [5] In-vitro biomechanical studies assessing the construct properties of plating versus retrograde screw fixation have shown better construct survivals and fracture fixation over time in superior ramus fractures treated with retrograde screws compared to those treated with plates. [6] Recently, a new percutaneous technique using an Elastic Stable Intramedullary Nail (ESIN) system for the treatment of superior ramus fractures has been proposed with suggested shorter operation times, less fluoroscopy times as well as increased one-time success when compared to retrograde screw fixation. [7] In-vitro biomechanical studies comparing the usage of ESIN compared to retrograde screw fixation are lacking and it was our hypothesis that elastic intramedullary nailing of pubic ramus fractures is as stable as retrograde screw fixation.

Thus, the purpose of this study was, to compare the biomechanical stability of elastic intramedullary nailing in pubic ramus fractures versus retrograde screw fixation.

## Methods

### Specimens

Eight fresh-frozen paired hemipelves (donors’ mean age 88 years, range 77 years to 100 years) were used for this biomechanical investigation. All remaining muscles were removed from the specimens, leaving only the bony components of each hemipelvis.

### Ethical considerations

All donors originated from the Institute of Anatomy of the University of Leipzig and had given written informed consent to dedicate their bodies to medical education and research purposes. Being part of the body donor program regulated by the Saxonian Death and Funeral Act of 1994 (3rd section, paragraph 18, item 8), institutional approval for the use of the post-mortem tissues of human body donors was obtained. The authors declare that all experiments were performed according to the ethical principles of the Declaration of Helsinki. Approval was obtained from the ethics committee of the Medical Faculty of Leipzig University (129–21-ek).

### Specimen preparation

**Fig. 1:**
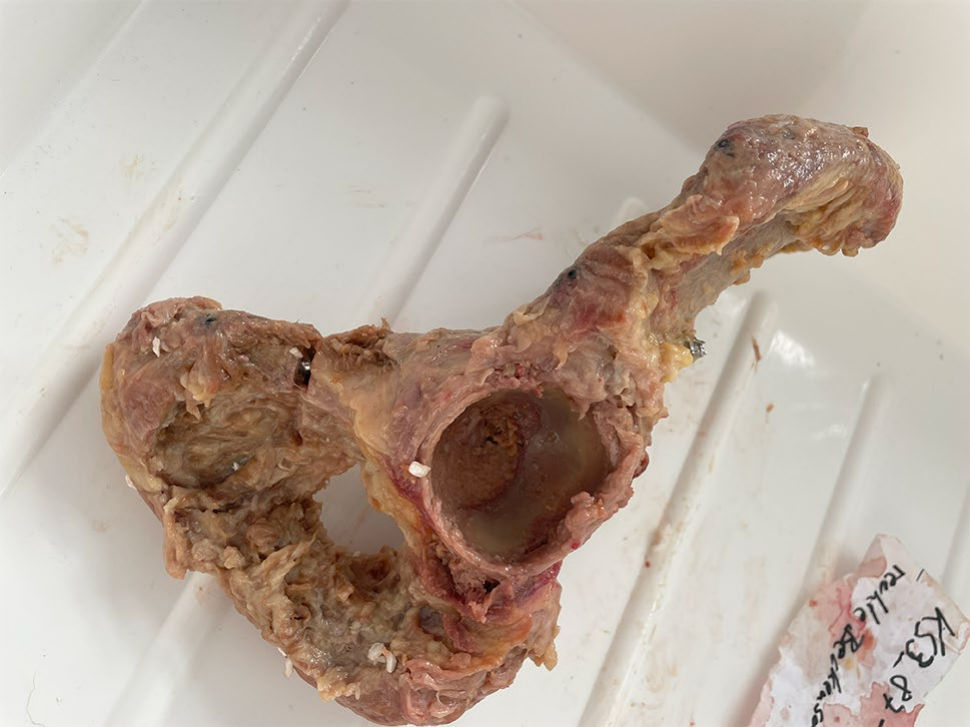
Prepared hemipelvis after fixation with a 6.5 mm cannulated ramus screw

**Fig. 2:**
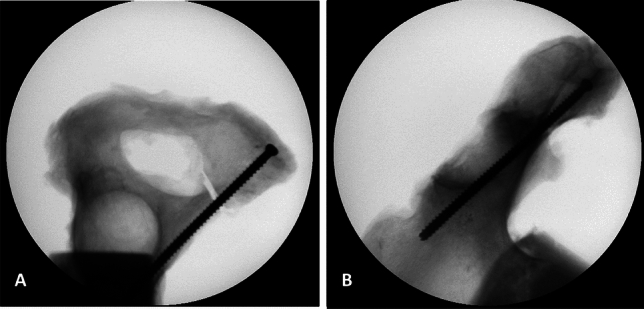
X-ray of superior ramus fracture stabilized with a 6.5 mm cannulated ramus screw; **A** Inlet-view; **B** Ala-view

**Fig. 3:**
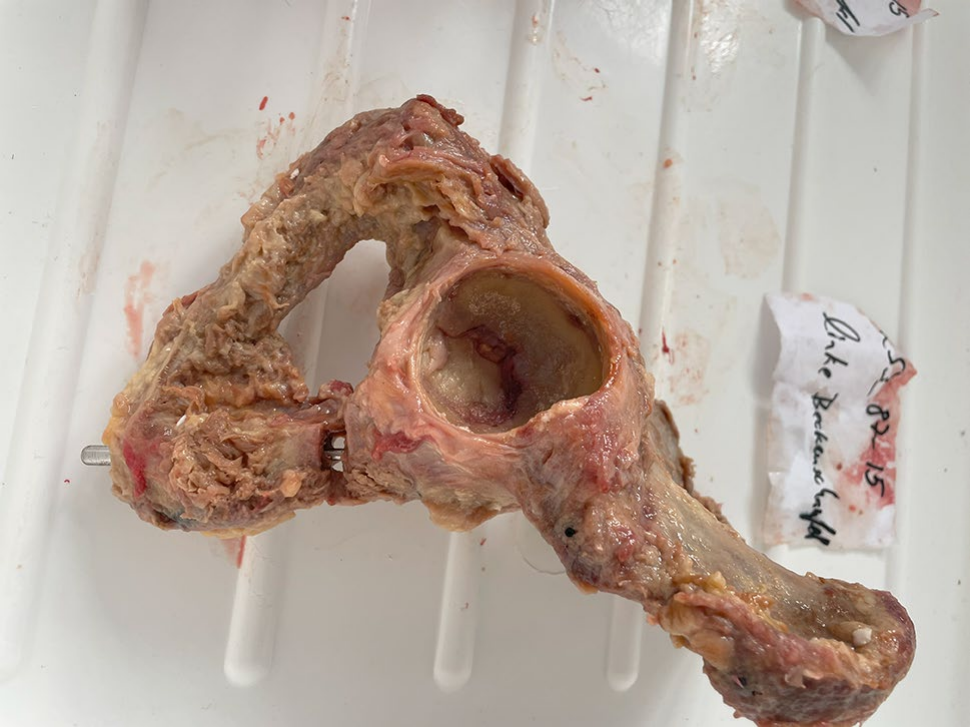
Prepared hemipelvis after fixation with a 6.5 mm cannulated ramus screw

**Fig. 4:**
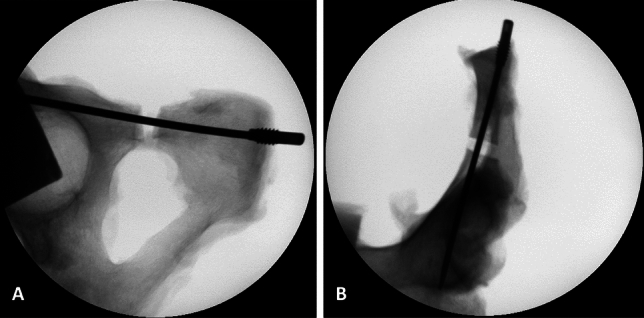
X-ray of superior ramus fracture stabilized with a 3.5 mm elastic intramedullary nail. **A** Inlet-view, **B** Ala-view

Specimen preparation and surgical fixation were performed by an experienced pelvis surgeon (GO) to ensure consistency. Standardized pubic ramus fractures were created by placing an osseous defect of 2 mm in the upper pubic ramus’ mid-portion (Nakatani type II) The pectineal ligament and the obturator membrane were removed. [8, 9] Of each pair of hemipelves one specimen was randomly assigned to one of two fixation techniques:

o Group 1: fracture stabilized by a retrograde 6.5 mm cannulated screw (length 150 mm, DePuySynthes, Solothurn, Switzerland) (Figs. 1,2).
o Group 2: fracture stabilized by a retrograde elastic intramedullary nail (3.5 mm, Stainless Steel Elastic Nail System, DePuySynthes, Solothurn, Switzerland) (Figs. 3,4).

All specimens were embedded in a mounting tray using high strength epoxy resin (RenCast® FC 52/53 Isocyanat, FC 53 Polyol, Huntsman Advanced Materials, Basel, Switzerland, mixed with Aluminiumhydroxide, Füller DT 082, Gößl + Pfaff GmbH, Karlskron/Brautlach, Germany), with the acetabular surface aligned parallel to the base plate.

### Biomechanical testing

The experimental setup was designed to progressively provoke implant loosening by fatigue or damage accumulation at the bone-implant interface. The samples underwent mechanical testing on a universal testing machine (AllroundLine Z010, ZwickRoell GmbH & Co KG, Ulm, Germany). A similar setup for pubic ramus fractures was used in a previous study. [10] Each specimen was subjected to consecutive cyclic loadings at 1 Hz with a sinusoidal axial force increasing from 100 to 750 N. After 500 cycles at 250 N, the axial load was increased to 400 N to simulate walking on a walker and after another 500 cycles, it was increased to 750 N to simulate full weight bearing. Before testing, all specimens were preloaded with 50 N for five cycles to level out initial subsidence. The force was applied in medio-superior direction through an endoprosthetic femoral head component on the acetabulum. [11]

**Fig. 5:**
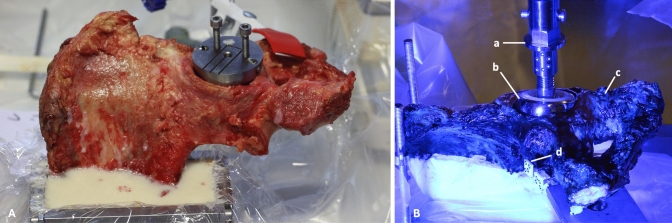
**A** embedded clinical specimen; **B** Testing set-up; a: AllroundLine Z010, ZwickRoell GmbH and Co KG; b: endoprosthetic femoral head component; c: specimen – hemipelvis; d: visual markers

The prepared specimens were fixed to a lockable x–y ground plate to allow specimen orientation before loading. The pubic symphysis was allowed to freely glide and pivot on the testing setup. (Fig. 5).

Axial displacement of the acetabulum beyond 25 mm (after pre-cycling) or any displacement at the fracture site beyond 10 mm was determined as failure resulting in an immediate stop of testing. In case of implant loosening or cut-out, testing was continued until definitive failure occurred (as determined previously).

Before specimen testing, the experimental setup as well as the biomechanical testing protocol was tested with a left Synbone hemipelvis (hemipelvis with acetabular diameter 54 mm, 4033, Synbone AG, Zizers, Switzerland). No limitations in the biomechanical setup and testing were found during pilot testing.

### Data acquisition

The movement at the fracture site was recorded using two visual markers attached 50 mm to each side of the fracture. The marker positions were tracked using an optical three-dimensional measurement system (ARAMIS 3D CAMERA, Carl Zeiss GOM Metrology GmbH, Braunschweig, Germany) (Fig. 5B). Before loading, one image frame was taken as a reference and during loading 100 image frames with a frame rate of 25 frames per second were taken over four loading cycles following every 100th cycle. Measurements were performed after 0, 100, 200, 300 and 400 cycles in each loading step. Recorded data was evaluated with the digital image correlation software GOM Correlate Pro (Carl Zeiss GOM Metrology GmbH, Braunschweig, Germany).

### Outcome variables

The primary outcome parameter was vertical fracture movement. Vertical fracture movement was defined as the difference in distance between the two markers in the y-axis. This was assessed by opto-metric analysis at the fracture site.

Secondary outcomes were horizontal and lateral fracture movement, construct survival and fracture motility over time. As a measure for accumulated fracture movement over time, the product of performed cycles and displacement was calculated and compared across the two groups using following formular: (D_I0_ + D_I100_ + D_I200_ + D_I300_ + D_I400_)*100 cycles + (D_II0_ + D_II100_ + D_II200_ + D_II300_ + D_II400_)*100 cycles + (D_III0_ + D_III100_ + D_III200_ + D_III300_ + D_III400_)*100. [12] [D_n,m_: Displacement at m cycles in loading stage m (I: 250N, II: 500N, III: 750N)].

### Statistical analysis

It was our hypothesis that fixation with an intramedullary elastic nail is not inferior to screw fixation. With an expected difference in fracture movement of 2 mm in both groups and a non-inferior margin of 2 mm, assuming a standard deviation of ± 2 mm, a type I error of α = 0.05 and a desired power of 0.80, a sample size calculation revealed a minimum sample size of 4 per group. [13]

Post-test analysis was done using SPSS for Mac V26.0 (IBM, Chicago, IL, USA). All data are reported as means with the standard deviation (SD). To assess differences in means between the two groups, an independent-samples t-test was used for normally distributed continuous data. To assess differences in means between the two groups, the Mann Whitney U test was used for not normally distributed continuous data (Shapiro–Wilk ≤ 0.05). The level of significance was defined as p < 0.05.

## Results

All specimens underwent the full testing protocol with 1500 cycles. A failure of the constructs was not observed in any of the samples.

### Y-axis

**Fig. 6:**
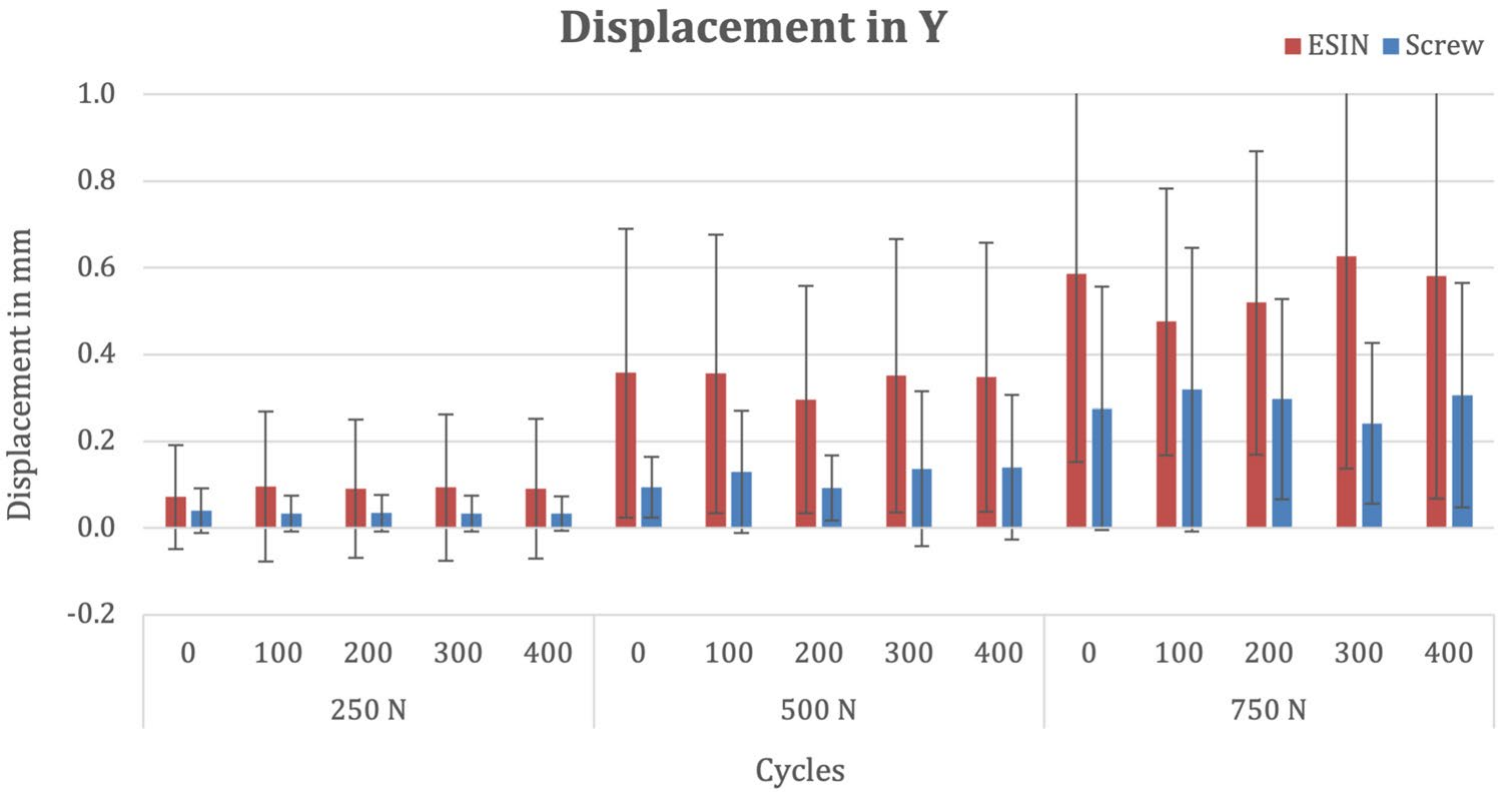
Bar chart depicting mean fracture displacement in the y-axis at various cycles and forces stratified by fixation method

There was no significant difference in mean vertical fracture displacement between the groups after osteosynthesis (Shapiro–Wilk ≤ 0.05; ESIN 0.07 mm, SD 0.12 versus screw 0.04 mm, SD 0.05; p = 0.773). After 400 cycles at 250 N, mean vertical fracture displacement (Shapiro–Wilk ≤ 0.05) was 0.09 mm (SD 0.16) in the ESIN group and 0.03 mm (SD 0.04) in the screw group (p = 0.773). After subsequent 400 cycles at 500 N in the vertical plane, mean fracture displacement increased to 0.35 mm (SD 0.31) in the ESIN group and to 0.14 mm (SD 0.17) in the screw group (p = 0.281). With a maximum load of 750 N, after 400 cycles, mean fracture displacement was 0.58 mm (SD 0.51) in the ESIN group and 0.31 mm (SD 0.26) in the screw group (p = 0.376). (Fig. 6).

There was no difference between the implants regarding the mean accumulated fracture movement over time (Shapiro–Wilk 0.135; ESIN 494 mm*cycles, SD 385 versus screw 220 mm*cycles, SD 210; p = 0.259).

### X-axis

**Fig. 7:**
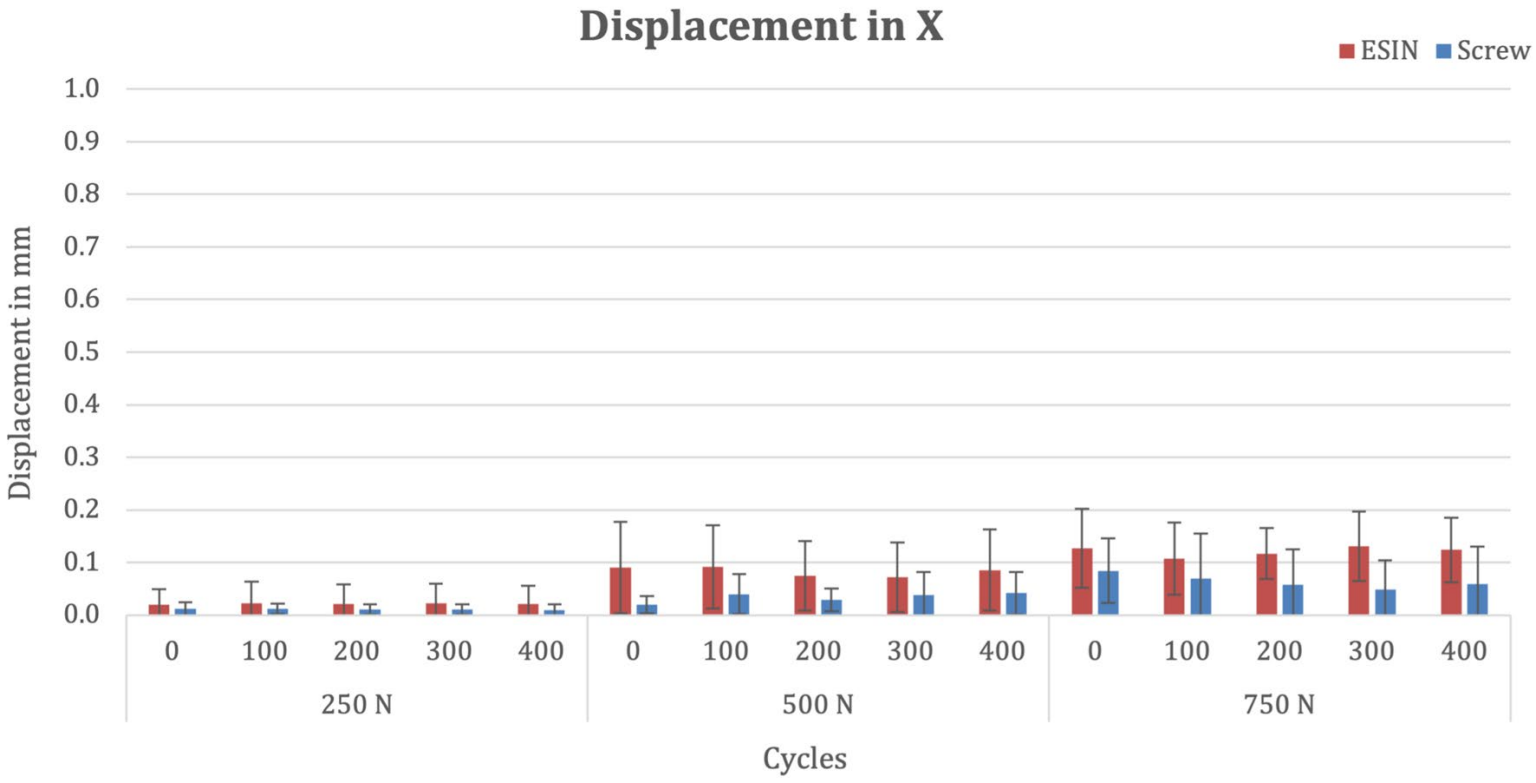
Bar chart depicting mean fracture displacement in the x-axis at various cycles and forces stratified by fixation method

There was no significant difference in mean horizontal fracture displacement between the groups after osteosynthesis ((Shapiro–Wilk ≤ 0.05; ESIN 0.019 mm, SD 0.03 versus screw 0.012 mm, SD 0.01; p = 0.773). After 400 cycles at 250 N, mean horizontal fracture displacement (Shapiro–Wilk ≤ 0.05) was 0.021 mm (SD 0.03) in the ESIN group and 0.01 mm (SD 0.01) in the screw group (p > 0.99). After subsequent 400 cycles at 500 N in the horizontal plane, mean fracture displacement increased to 0.085 mm (SD 0.08) in the ESIN group and to 0.042 mm (SD 0.04) in the screw group (p = 0.353). With a maximum load of 750 N, after 400 cycles, mean fracture displacement was 0.124 mm (SD 0.06) in the ESIN group and 0.059 mm (SD 0.07) in the screw group (p = 0.218). (Fig. 7).

There was no difference between the implants regarding the mean accumulated fracture movement over time (Shapiro–Wilk 0.076; ESIN 113 mm*cycles, SD 77 versus screw 54 mm*cycles, SD 52; p = 0.255).

### Z-axis

**Fig. 8:**
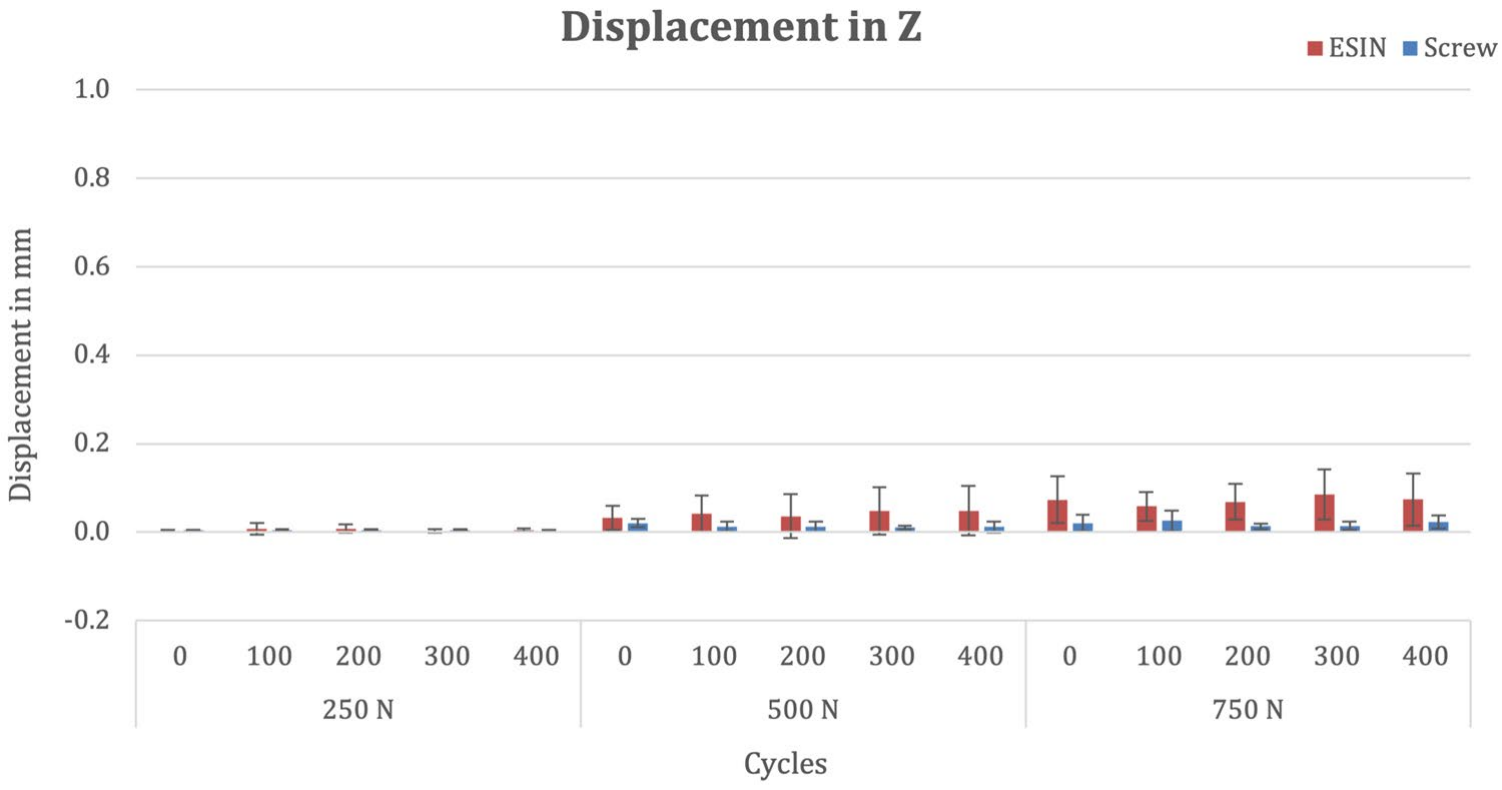
Bar chart depicting mean fracture displacement in the z-axis at various cycles and forces stratified by fixation method

There was no significant difference in mean lateral fracture displacement between the groups after osteosynthesis (ESIN 0.03 mm, SD 0.03 versus screw 0.004 mm, SD 0.001; p = 0.357). After 400 cycles at 250 N, mean lateral fracture displacement was 0.005 mm (SD 0.003) in the ESIN group and 0.003 mm (SD 0.003) in the screw group (p = 0.211). After subsequent 400 cycles at 500 N in the lateral plane, mean fracture displacement (Shapiro–Wilk ≤ 0.05) increased to 0.049 mm (SD 0.056) in the ESIN group and to 0.012 mm (SD 0.012) in the screw group (p = 0.248). With a maximum load of 750 N, after 400 cycles, mean fracture displacement (Shapiro–Wilk ≤ 0.05) was 0.073 mm (SD 0.06) in the ESIN group and 0.059 mm (SD 0.023) in the screw group (p = 0.149). (Fig. 8).

There was no difference between the implants regarding the mean accumulated fracture movement over time (Shapiro–Wilk 0.043; ESIN 59 mm*cycles, SD 44 versus screw 19 mm*cycles, SD 11; p = 0.114).

## Discussion

Due to the minimal invasiveness and proven biomechanical stability, fixation of superior ramus fractures using cannulated retrograde screws, has become a well-accepted fixation option in the treatment of these fractures. [5, 6] Elastic intramedullary nailing (ESIN) has been introduced by Jean Prevot and Jean-Paul Metaizeau in the late 1970s, and has become a key option in the treatment of paediatric long-bone fractures. [14] However, ESIN was shown to be superior to conventional plating in certain assorted fractures of the adult skeleton with good functional outcome as well. [15] Recently, the use of ESIN has been proposed for the treatment of superior ramus fractures. [7] Proposed indications for the use of ESIN in these fractures are a very narrow osseous canal, a very curved superior ramus and the osteoporotic patient due to reduced concentrated stress to the bone and less likely cut-out into the pelvis compared to rigid ramus screws. [16, 17] Further it has been clinically shown, that ESIN had less intraoperative fluoroscopic time, less operative time and a higher one-time success rate compared to retrograde ramus screws. [7] Further, a recent case series of 19 patients with pelvic ring fractures treated with titanium elastic nail (TEN) showed 100% union rate and very low complication rates suggesting high feasibility of this technique for the treatment of pelvic ring injuries. [18] A cyclic loading biomechanical comparison of ESIN and retrograde ramus screw, to the authors` knowledge, has not yet been conducted. Thus, the aim of this study was, to compare the biomechanical stability of ESIN in pubic ramus fractures versus retrograde screw fixation.

In this in vitro study, the biomechanical stability between ESIN and retrograde ramus screw was assessed in three planes (y,x,z-axis). We did not observe any construct failures over 1500 cycles with a maximum force of 750N, which is in keeping with previous biomechanical studies on retrograde ramus screw fixation. [6, 19] The intact ESIN-construct in our study therefore suggests equal stability compared to the ramus screw. We did not assess any statistically significant difference in regard to fracture displacement in any plane between the two implants, which suggests good fixation with both, ESIN and ramus screw. However, the ESIN hemi-pelves showed generally higher standard deviation as well as mean fracture displacement over time. This finding is in keeping with a study from China, where they assessed marginally inferior (statistically not significant) biomechanical properties with ESIN compared to retrograde ramus screw. [7] This study only assessed biomechanical properties by applying 500N to the S1 vertebral endplate and that artificial bone models were used. We further assessed no statistically significant differences between the assessed implants regarding mean absorbed energy over time in all planes. Our findings support the reportedly good outcomes using ESIN for the fixation of Nakatani type II and III fractures in a small case series from Mexico as well as in a study from China which should very good outcomes without any complication in a series of 24 patients. [17, 20]

### Limitations

We did not assess the bone density of the chosen hemi-pelves which might have differed between the implants. However, to reduce this effect, we have assigned one hemi-pelvis from the same donor to each group. The cadaver model used did also not account for muscle forces acting on the anterior pelvic ring in vivo. During walking, load patterns may significantly change in intensity and direction. This cannot be simulated by an in vitro setup. The bone defects made included the pectineal ligament that serves as secondary stabilizer of minimally displaced pubic ramus fractures. [10] Hence, the setup used in this study is a worst-case-scenario and it well be possible that the displacement observed in vivo might be even less in most cases. Further, we did not assess Nakatani type I fractures, which are known to have a poorer outcome with retrograde ramus screws compared to Nakatani type II and III fractures. [8] Finally, we tested a 6.5 mm retrograde ramus screw which is clinically not always possible to use, hence many surgeons prefer 3.5 mm screws.

## Conclusions

Based on the results of this in vitro study, there was no biomechanical difference between the conventional percutaneous retrograde pubic ramus screw and the percutaneous ESIN system. Thus, it seems that ESIN is a viable alternative device for the treatment of superior ramus fractures. In vivo studies are needed to confirm these findings.

## Data Availability

Data is available upon reasonable request to the corresponding author.
